# Protocol for determining protein cysteine thiol redox status using western blot analysis

**DOI:** 10.1016/j.xpro.2021.100566

**Published:** 2021-06-09

**Authors:** Bikram Datt Pant, Sunhee Oh, Kirankumar S. Mysore

**Affiliations:** 1 Noble Research Institute, 2510 Sam Noble Parkway, Ardmore, OK 73401, USA; 2 Institute for Agricultural Biosciences, Oklahoma State University, Ardmore, OK, 73401, USA; 3 Department of Biochemistry and Molecular Biology, Oklahoma State University, Stillwater, OK, 74078, USA

**Keywords:** Plant sciences, Molecular Biology, Protein Biochemistry

## Abstract

This protocol describes the analysis of protein cysteine redox status. Redox status is crucial in regulating protein activity, stability, and redox signaling cascades. It is determined by conjugation with 1.24 kDa MM(PEG)_24_ molecule to each reduced cysteine followed by western blot analysis. This protocol is easy to follow, and most of the reagents and instruments required are of common use in any lab. This protocol can be successfully applied to other biological sources.

For complete details on the use and execution of this protocol, please refer to Pant et al. (2020).

## Highlights

• Protein redox status is crucial in regulating its activity, stability, and signaling
• MM(PEG)24 reacts with reduced cysteine, increasing protein molecular weight by 1.24 kDa
• Protein’s reduced status is determined by increased molecular weight in western blot

## Graphical abstract

**Figure:**
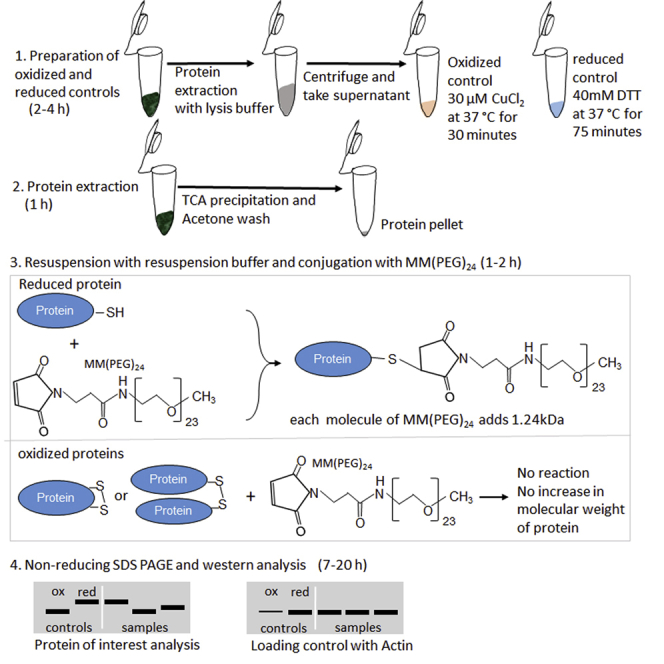

## Before you begin

The protocol below describes the specific steps for detection of reduced or oxidized status of protein cysteine-thiols. We have used this protocol to determine the redox state of Thioredoxin like 1 (TRXL1) protein in plants grown under normal condition or subjected to abiotic and biotic stress for various time points (Pant et al., 2020). Oxidation-reduction of regulatory proteins and metabolic enzymes containing cysteine thiols leads to formation or breakage of disulfide bond causing changes in protein conformation, folding, monomerization or oligomerization or complex formation (Chung et al., 2013; Rouhier et al., 2015; Waszczak et al., 2015). Protein thiol redox modification alters protein activity, stability and redox signaling cascades in cell under normal condition or in response to various factors such as diurnal, environmental and developmental cues (Brandes et al., 2009; Finkel, 2011; Putker and O'Neill, 2016).

### Generating biological samples as a protein source

**Timing: 1 day to 1 month**

Detection of the protein by western blot analysis will require an antibody against the target protein. If the antibody is not available, target protein will need to be tagged. We suggest using a small epitope tag that does not contain cysteine such as HA (YPYDVPDYA), FLAG (DYKDDDDK) or Myc (YPYDVPDYA). The reason for using short tag is to decrease the number of external amino acids added that may sometimes interfere with protein expression or folding. Absence of cysteine in the tag is to avoid its possibility of being labeled with MM(PEG)_24_ that might generate false results. Grow biological material expressing the protein of interest and harvest under frozen conditions in liquid nitrogen.

### Preparation of buffers and chemicals

**Timing: 2–4 h**

1. 10% (v/v) Trichloroacetic acid (TCA). Keep it at 4°C to make it cold before use.
2. 90% (v/v) Acetone in water. Cool the 90% and 100% acetone at −20°C before use.
3. 10 mM stock of the methyl-maleimide polyethylene glycol [MM(PEG)_24_] in dimethylformamide (DMF) or dimethylsulfoxide (DMSO). Make aliquots of stock solution to avoid freeze-thaw and store at −20°C in moisture free condition.

**Table:** Lysis buffer

Reagents	Stock (prepare individually)		Final concentration	Amount (1×)
Tris, pH 7.5	121.14 g	1M	50mM	2.5 mL
NaCl	292.2 g	5M	150 mM	1.5 mL
MgCl_2_	406.62 g	2M	10 mM	0.25 mL
Glycerol	**n/a**	100%	10%	5 mL
NP40	**n/a**	20%	0.1%	0.25 mL
EDTA	186.12 g	0.5M	1 mM	0.1 mL
PMSF	17.419 g	0.1M	1 mM	0.5 mL
Water	1 L	**n/a**	**n/a**	39.9 mL
**Total**	**1 L**	**n/a**	**n/a**	**50 mL**

Store at 4°C. Store PMSF and protease inhibitor cocktail solution at −20°C and is stable for several months. Add 1×protease inhibitor cocktail just before use.

**Table:** Resuspension buffer

Reagents	Stock (20×)		Final concentration	Amount (1×)
Tris, pH 7.5	48.5 g	400 mM	50 mM	6.25 mL
Urea	n/a	n/a	4 M	12.01g
Glycerol	n/a	n/a	2.5%	1.25 mL
SDS	n/a	n/a	2%	1 g
Bromophenol blue	n/a	n/a	0.005%	2.5 mg
Water	1 L	n/a	n/a	upto 50 mL
**Total**	**1 L**	n/a	n/a	**50 mL**
**PH**	n/a	n/a	n/a	**7**

It is better to use freshly added urea in the solution as solutions of urea can produce reactive cyanate ions upon standing. Buffer without urea can be stored at 4°C to 25°C for more than a year.

**Table:** Running buffer

Reagents	Stock (10×)		Final concentration	Amount (1×)
Tris	30.28 g	250 mM	25 mM	100 mL of Tris, Glycine, SDS mix
Glycine	142.63 g	1.9 M	190 mM	
SDS	10 g	1%	0.1%	
Water	1 L	n/a	n/a	900 mL
**Total**	**1 L**	n/a	n/a	**1 L**

Buffer can be stored at 4°C to 25°C for more than a year.

**Table:** Transfer buffer

Reagents	Stock (10×)		Final concentration	Amount (1×)
Tris	30.28 g	250 mM	25 mM	100 mL of Tris, Glycine mix
Glycine	142.63 g	1.9 M	190 mM	
Methanol	n/a	n/a	20%	200 mL
Water	1 L	n/a	n/a	700 mL
**Total**	**1 L**	n/a	n/a	**1 L**

Add methanol just before use. Buffer without methanol can be stored at 4°C to 25°C for more than a year.

**Table:** Tris-buffered saline with Tween 20 (TBS-T) buffer

Reagents	Stock (20×)		Final concentration	Amount (1×)
Tris, pH 7.6	48.5 g	400 mM	20 mM	50 mL of Tris and NaCl mix
NaCl	175.32 g	3 M	150 mM	
Tween 20	n/a	n/a	0.1%	1 mL
Water	1 L	n/a	n/a	949 mL
**Total**	**1 L**	n/a	n/a	**1 L**

Buffer can be stored at 4°C to 25°C for more than a year.

For hybridization with antibody, use freshly prepared 1% non fat dry milk in TBS-T.

**Table:** Stripping buffers

Reagents	Stock (20×)		Final concentration	Amount (1×)
Tris, pH 6.8	60.57 g	0.5M	62.5 mM	3.75 mL 3 mL
SDS	200 g	20%	2%	
β-mercaptoethanol	n/a	14.3M	100 μM	210 μL
Water	n/a	n/a	n/a	upto 30 mL
**Total**	**1 L**	n/a	n/a	**30 mL**

Buffer can be stored at 4°C to 25°C for more than a year. Add β-mercaptoethanol just before use.

## Key resources table

**Table:** 

REAGENT or RESOURCE	SOURCE	IDENTIFIER
**Antibodies**		

Anti-HA antibody	https://www.abcam.com	Cat#ab9110; RRID:AB_307019
Anti-Actin antibody	Sigma-Aldrich	Cat#A0480; RRID:AB_476670
Goat Anti-Rabbit IgG H&L	https://www.abcam.com	Cat#ab6721; RRID:AB_955447

**Biological samples**		

AtTRXL1-HA expressing Arabidopsis lines	Pant et. al, 2020; Mysore Lab, Noble Research Institute	N/A

**Chemicals, peptides, and recombinant proteins**		

Methyl-maleimide polyethylene glycol MM(PEG)_24_	Thermo Fisher Scientific	Cat#22713
4–15% Criterion TGX Stain-Free Protein Gels	Bio-Rad	Cat#5678083
Pierce™ ECL Western Blotting Substrate	Thermo Fisher Scientific	Cat# 32209
10× Tris/Glycine/SDS Running Buffer	Bio-Rad	Cat #1610772
Trichloroacetic acid (TCA)	Sigma	Cat# T6399
Protease inhibitor cocktail	Sigma	Cat#P9599
Blotting Grade Blocker Non Fat Dry Milk	Bio-Rad	Cat #1706404XTU
CuCl_2_	Sigma-Aldrich	Cat#203149-10G
DTT (dithiothreitol)	Thermo Fisher Scientific	Cat# R0861
E-Z Store & Pour Fixer	MXR, The Imaging Solution Company	Cat# 114511
E-Z Store & Pour Developer	MXR, The Imaging Solution Company	Cat# 103633
Acetone	Sigma-Aldrich	Cat# 179124-500ML

**Software and algorithms**		

ImageJ	Rueden et al., 2017, https://imagej.net/Welcome	N/A

**Other**		

Criterion Vertical Electrophoresis Cell	Bio-Rad	Cat#1656001
Trans-Blot SD Semi-Dry Transfer Cell	Bio-Rad	Cat# #1703940
Nitrocellulose membrane	Bio-Rad	Cat#1620112
X-Ray Film	Thomas Scientific	Cat#1148B77
Konica medical film processor SRX-101A	Konica Corporation	Cat# 1051/1052
Aluminum Push Button X-ray Cassette - 8×10	soyeeproductsny.com	N/A

## Step-by-step method details

**Timing: 2–3 days**

### Preparation of oxidized and reduced controls

**Timing: 2–4 h**

1. Grind frozen biological material and aliquot equal amount (e.g., 20 mg) for each sample in 1.5 ml tubes.
2. Add 90 μL lysis buffer, vortex mix and incubate onto ice for 15 min.
3. Centrifuge at maximum speed for 15 min then transfer the supernatant into new tube.
4. For preparing oxidized control, add 10 μL CuCl_2_ (from 10× stock of 300 μM) into 90 μL protein extract to a final concentration of 30 μM and incubate at 37°C for 30 min. Caution: CuCl_2_ is considered hazardous by the Occupational Safety and Health Administration (OSHA) Hazard Communication Standard.
5. For preparing reduced control, add 10 μL DTT (from 10× stock of 400 mM) into 90 μL protein extract to a final concentration of 40 mM and incubate at 37°C for 75 min.

**Pause point:** oxidized and reduced samples can be stored at −20°C.

### Protein extraction and conjugation with MM(PEG)_24_

**Timing: 3–4 h**

This step involves extraction of protein from biological materials and conjugation of reduced protein cysteine thiols with MM(PEG)_24_. Addition of cold TCA into frozen biological materials denatures and precipitates proteins. It quenches the thiol oxidation, and native redox state of cysteine thiol is preserved (Hansen et al., 2013). Treatment of proteins with MM(PEG)_24_ adds a molecule of MM(PEG)_24_ to each reduced cysteine thiol thereby increasing the molecular weight of the protein by 1.24 kDa for each molecule added. This increased molecular weight of the protein is the basis of determination of reduced state of protein cysteine thiols.

6. Grind biological materials in liquid nitrogen and aliquot 20 mg each in 1.5 mL tubes. The amount of biological material can be optimized based on protein expression levels.
7. Add 1 mL of cold 10% (v/v) TCA to samples, and to the oxidized and reduced controls. Mix immediately by vortexing.
8. Incubate in ice for 30 min.
9. Centrifuge at 16,000 g at 4°C for 10 min.
10. Wash and dry the pellets. Add 1 mL of cold 90% acetone, vortex mix, centrifuge at 11000 g for 5 min and remove the supernatant. Repeat the wash with 1 mL of cold 100% acetone. Dry the pellet at 37°C for 5 min. Do not over dry.
11. Resuspend the pellets in 90 μL of resuspension buffer. Add 10 μL of 10 mM MM(PEG)_24_. Resuspension buffer contains SDS that will denature proteins exposing cysteine thiols.
12. Incubate at 65°C for 5 min and then at 22°C to 25°C for 1 h for protein thiol alkylation. Incubation at 65°C will help to denature the proteins exposing the cysteine thiols. Reaction with MM(PEG)_24_ results in formation of stable, irreversible thioether bond with cysteine thiols.

**Pause point:** samples can be stored at 4°C for about a week. It is better to run as fresh as possible.

### Running non-reducing SDS (sodium dodecyl sulfate)-PAGE (polyacrylamide gel electrophoresis) and western blot analysis

**Timing: 7–20 h**

This step accomplishes the fractionation of proteins by SDS-PAGE, the transfer of proteins into a membrane, imaging and data analysis. Detection of increased molecular weight determines the cysteine thiol reduced state of protein.

#### Running non-reducing SDS-PAGE

**Timing: 1.5–2 h**

13. Load gels in the gel-tank and fill it with running buffer.
14. Load equal amount (10–40 μL) of each sample into well.
15. Run the gel at 120 V until the bromophenol dye reaches to the bottom of the gel.
16. Take out the gel from gel tank, rinse the cassette with sterile distilled water, open the gel-cassette and put the gel in a tray with 1× transfer buffer.

#### Transferring protein from the gel into the membrane using semi-dry transfer cell

**Timing: 1–2 h**

17. Soak two extra thick blotting papers and one nitrocellulose membrane (similar to the size of gel) in the transfer buffer.
18. In semi-dry transfer cell, assemble the transfer sandwich in the following order: blotting paper, membrane, gel, and blotting paper. Remove any air bubbles and make sure no air bubbles are trapped in the layers. Alternatively, wet transfer system can be used.
19. Transfer at 15–25 V for 30–40 min for midi-gels for proteins of 25 kDa to 150 kDa.

Gel blot conditions for trans-blot semidry system can be optimized as follows.

For midi gels: 15V–25V/3 mA/cm^2^ for 30–40 min. For large gels: 15–25 V/ 3 mA/cm^2^ for 60 min. We used extra thick absorbent blotting paper on both side because of its higher buffer absorbing capacity. Setting up a test run to determine the time for complete protein transfer will be helpful if the settings suggested here do not work. Protein transfer from gel to membrane may take from 10 min to 16 h depending on the power settings, gel percentage, and size of the protein. Transfer of high molecular weight protein will need longer time and vice-a-versa. Check if the marker proteins are transferred well to determine the complete transfer of target protein.

#### Protein hybridization with antibody

**Timing: 4–16 h**

20. Rinse the membrane with TBS-T. Block the membrane with 5% skimmed milk in TBS-T for 1 h at 22°C to 25°C with gentle shaking.
21. Rinse the membrane with TBS-T. Incubate with the primary antibody against the target protein in 1% skimmed milk in TBS-T at 4°C for 12 to 16 h or at 22°C to 25°C for 1 h. We used anti-HA antibody for TRXL1-HA protein at 1:10000 dilution in 1% skimmed milk in TBS-T. TRXL1 is a thioredoxin like chloroplastic drought induced stress protein (CDSP32) encoded by *At1g76080**.*
22. Wash the membrane 3–4 times for 15 min each with TBS-T with gentle shaking.
23. Incubate with HRP conjugated secondary antibody at 22°C to 25°C for 1 h. We used Goat Anti-Rabbit IgG H&L at 1:20000 dilution in 1% skimmed milk in TBS-T.

***Note:*** Choose your secondary antibody based on the source of primary antibody. Our antibody was developed in rabbit, so we used anti-rabbit antibody.

24. Wash the membrane 3–4 times for 15 min each with TBS-T.

#### Imaging and data analysis

**Timing: 1 h**

Prepare the ECL western blotting detection reagent by mixing equal amount of solution A and B provided by the manufacturer. Approximately 2–3 mL solution is required for a membrane of 12 cm × 6 cm.

25. Remove excess buffer from membrane, do not let it dry. Place membrane in tray, apply ECL solution, and incubate at 22°C to 25°C for 0.5–5 min.
26. Remove excess ECL solution.
27. Keep membrane inside plastic wrap inside X-ray film cassette. Expose membrane to the X-ray film in cassette in dark room for few seconds to hours depending the signal intensity.
28. Develop the x-ray film. We developed with the help of Konica medical film processor using E-Z Store & Pour fixer and E-Z Store & Pour developer. Alternatively, manual processing can be done.
29. Measure the band intensity using image analysis software such as ImageJ (Rueden et al., 2017). Normalize the band intensity with a housekeeping protein like Actin. Calculate the relative intensity of bands compared to a control.

#### Loading control

The same membrane is stripped and re-probed with housekeeping protein antibody. We used anti-Actin antibody to detect Actin as loading control as it is a housekeeping protein.

30. Incubate membrane in stripping buffer at 50°C for 30 min with gentle shaking. Wash the membrane with TBS-T three times every 15 min.
31. Reprobe with primary antibody against Actin as explained above from step # 20 to 29.

## Expected outcomes

**Figure 1:**
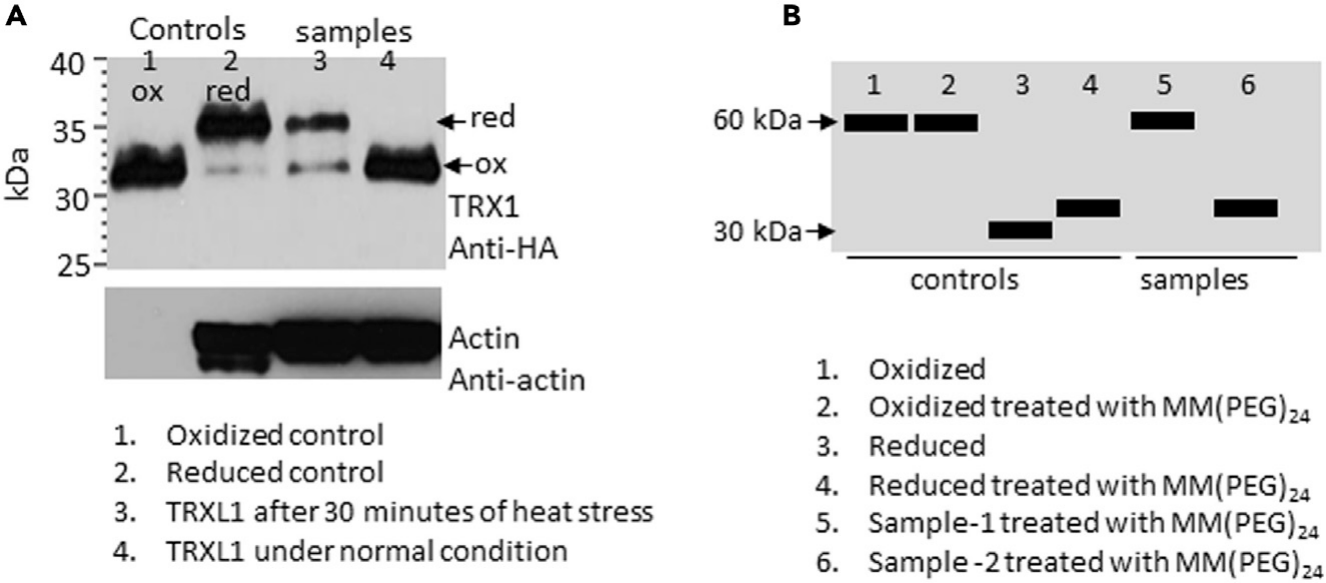
Analysis of protein cysteine thiol redox status (A) Redox assay western blot analysis of *Arabidopsis thaliana* expressing AtTRXL1-HA protein. Lane 1 and 2 shows the oxidized (ox) and reduced (red) controls, respectively. Lane 3 shows the TRXL1-HA from plants subjected to heat stress at 40°C for 30 min and Lane 4 from plants grown under normal condition at 22°C. Conjugation of MM(PEG)_24_ to reduced cysteine thiols led to an increased molecular weight of TRXL1 in reduced control (lane 2) and heat stressed sample (lane 3). Protein size scale is given in kDa. Membrane was stripped and re-probed with anti-Actin antibody as a loading control. Actin is not visible in oxidized lane possibly because the oxidation of Actin may lead to the actin polymerization. (B) Graphical illustration of oxidized and reduced state of dimeric protein showing controls and samples.

Non-reducing western blot analysis of MM(PEG)_24_ treated proteins show if a protein of interest is in a oxidized or reduced state. The reduced state of protein is marked by an increase in their molecular weight (Figure 1A, Lanes 2 and 3) due to the addition of MM(PEG)_24_ molecules compared to oxidized (Figure 1A, Lanes 1 and 4). Some proteins exist as oligomers in normal or in oxidized conditions by formation of disulfide bonds between monomers. Under reduced state, these become monomers due to breakage of disulfide bonds between oligomers. In such situation, band size of the reduced protein will appear smaller (Figure 1B, Lanes 3, 4 and 6) than oxidized (Figure 1B, Lanes 1, 2 and 5) in western blot. This can be determined by comparing the samples (Figure 1B, Lanes 5 and 6) with oxidized control and reduced controls that are untreated or treated with MM(PEG)_24_ (Figure 1B, Lane 1–4). The size of the reduced protein will be smaller than oxidized by oligomerization factor. For example, a 60 kDa dimerized protein will have approximately 30 kDa of reduced monomer without MM(PEG)_24_. Reduced protein treated with MM(PEG)_24_ will have an increased molecular weight (Figure 1B, lane 4 and 6) compared to reduced untreated (Figure 1B, lane 3).

## Limitations

Oxidation of some protein thiols may lead to the formation of polymer or complex of large molecular weight. These large complexes cannot be electrophoresed and hence not visible in western blot.

Protein cysteine can be in reduced state or oxidized to S-nitrosocysteine, cysteine sulfenic and sulfinic acids, disulfides and persulfides (Alcock et al., 2018). This assay addresses the presence of reduced cysteine as MM(PEG)_24_ reacts with reduced cysteine. For labeling of oxidized protein thiols, an approach (Rudyk and Eaton, 2014) is given as follows:- (i) reduced thiols are treated with an alkylating reagent (maleimide) that adds maleimide to reduced thiol and block it. (ii) Oxidized thiols are reduced with reducing agents such as DTT. (iii) Newly formed reduced thiols are labeled with labeling agent such as botin-malemide.

## Troubleshooting

### Problem 1

Protein band intensity in the gel-blot too strong or too weak (step 28)

### Potential solution

Optimize the amount of starting biological material.

Adjust the amount the protein loaded in the gel.

Decrease or increase the time of exposure of the gel-blot to X-ray film.

### Problem 2

No increase in the molecular weight of reduced protein (step 28).

### Potential solution

If you expect your protein is in reduced state but do not see an increased molecular weight after conjugation with MM(PEG)_24_ :

Make sure that the conjugation with MM(PEG)_24_ is working well by comparing it with control reduced sample as a reference.

Use fresh or −20°C stored aliquots of MM(PEG)_24_.

### Problem 3

Separation between the reduced MM(PEG)_24_ treated and oxidized protein is very little (step 28).

### Potential solution

We suggest using 4%–15% or 4%–20% gradient gel instead of normal gel for better separation of proteins.

### Problem 4

Smear in protein band (step 28)

### Potential solution

It can happen because of the protein degradation.

Make sure to use fresh/frozen protease inhibitor cocktail in lysis buffer.

Extract the protein under cold condition to avoid degradation.

Use the freshly prepared protein extract for western gel blot without storing it for longer period.

### Problem 5

Small size protein not appeared on the gel-blot (step 28)

### Potential solution

Make sure that it did not run out of gel during electrophoresis.

Decrease the duration and/or voltage of transfer from gel into membrane as smaller protein will move faster or may pass out through the membrane.

## Resource availability

### Lead contact

Further information and requests for resources and reagents should be directed to and will be fulfilled by the lead contact, Kirankumar S. Mysore (ksmysore@noble.org).

### Materials availability

This study did not generate new unique reagents.

### Data and code availability

This study did not generate new datasets.
